# Preliminary assessment of neck circumference in benign prostatic hyperplasia in patients with metabolic syndrome

**DOI:** 10.1590/S1677-5538.IBJU.2016.0139

**Published:** 2017

**Authors:** Yigit Akin, Hakan Gulmez, Erhan Ates, Mehmet Gulum, Murat Savas

**Affiliations:** 1 Department of Urology, Harran University School of Medicine, Sanliurfa, Turkey;; 2 Department of Family Medicine, 14th Family Healthcare centre, Konya, Turkey;; 3 Department of Urology, Necip Fazil State Hospital, Kahramanmaras, Turkey;; 4 Department of Urology, Hacettepe University School of Medicine, Ankara, Turkey;; 5 Department of Urology, Antalya Training and Research Hospital, Antalya, Turkey

**Keywords:** Prostatic Hyperplasia, Quality of Life, Patients

## Abstract

**Objectives:**

To investigate the impact of neck circumference (NC) in the treatment of bening prostatic hyperplasia (BPH) patients with metabolic syndrome (MtS). Additionally, we determined dose response to alpha-blockers and cut-off values for NC and waist circumference (WC), in these patients.

**Materials and Methods:**

Non-randomized, open-labelled, and multi-centre study was conducted between March 2014 and September 2015. The BPH patients were enrolled and were divided into 2 groups: with MtS (Group 1; n=94) and without MtS (Group 2; n=103). Demographic data, anthropometric measurements, blood analyses, uroflowmetric parameters, post voiding residual urine (PVR), prostate volume, quality of life (QoL) index, NC and WC were recorded. Both groups were administered oral alpha-blockers and response to treatment was evaluated. Receiver-operating characteristic (ROC) curves were obtained and significant p was p<0.05 *.*

**Results:**

In total, 197 patients were enrolled with mean age of 60.5±8.1 years. Mean NC and WC were higher in MtS patients (p<0.001). Uroflowmetry parameters and QoL indexes were comparable between groups before treatment. International prostate symptom score, uroflowmetry parameters, and QoL significant improved in Group 2 than Group 1, at 1 ^st^ and 6 ^th^ months of treatment with alpha-blockers. Success rate of treatment was significant higher in Group 2 than Group 1 (p<0.001). Cut-off values were 42.5cm and 113.5cm for NC and WC respectively, for response to alpha-blockers in BPH patients with MtS.

**Conclusions:**

MtS can be related with BPH and can negatively affect the response to alpha-blocker treatment. NC can be used for predicting response to alpha-blocker treatment in BPH patients with MtS.

## INTRODUCTION

Benign prostatic hyperplasia (BPH) is one of the most frequent diseases in aging men ( 1 ). Alpha-blockers are the first choice of medical treatment of BPH ( 2 ). However, it is still controversial, which patient profile would respond to the alpha-blockers or not ( 3 ). Additionally, accurate doses of alpha-blockers are still unknown, in case of comorbidities such as metabolic syndrome (MtS) ( 4 ). Recently, it was established that MtS is one of the causing factors for the development of BPH in aging men ( 5 ).

MtS consists of some metabolic risk factors on individuals ( 6 ). However, there are several descriptions for MtS, and the criteria of The National Cholesterol Education Program (NCEP) Expert Panel on Detection, Evaluation, and Treatment of High Blood Cholesterol in Adults (Adult Treatment Panel III) (ATP III) is the mostly used ( 7 ). According to this guideline, the presence of three of the following risk factors constitute MtS: blood pressure (BP) ≥130/85mmHg, fasting blood glucose (FBG) ≥110mg/dL, waist circumference (WC) ≥102cm, High-density lipoprotein (HDL)-cholesterol <40mg/dL, serum triglicerides (TG) ≥150mg/dL, in one individual. Central/visceral obesity is associated with MtS due to visceral adipose tissue ( 8 ). According to NCEP, WC is an indicator of central obesity as well as of visceral adipose tissue ( 7 ). The measurement of WC sometimes could be very difficult, time consuming, and an inaccurate measurement could be performed in outpatient clinics. Thus, another easy applicable method of measuring visceral obesity is necessary. The neck circumference (NC) comes into question at this point ( 9 ). Previous studies showed the usefulness of NC for determining visceral obesity ( 10 , 11 ). According to our best knowledge, there is no published study on relationship between NC and BPH in patients with MtS.

We here investigate anthropometric details of BPH patients with MtS: NC and WC and additionally, determined cut-off values of NC, WC to predict response to alfa-blocker treatments.

## MATERIALS AND METHODS

### Study design

This is a prospective, non-randomized, multi-centre, and open-labelled study. Between March 2014 and September 2015, BPH patients who were admitted at family medicine and urology outpatient clinics were evaluated. Our study was approved by instituonal review board. All patients understood aims of the study and also signed consent forms including standards of the 2008 Helsinki declaration and its later amendments or comparable ethical standards. The exclusion criteria were: previous prostate surgery and/or any prostate disease, prostate specific antigen (PSA) >4ng/dL, suspicious prostate nodule in digital rectal examination (DRE), urinary infections, any neurologic disease, allergy to any alpha-blockers, and any bladder disease.

### Data collection

247 symptomatic BPH patients were included. All patients were administered non-randomized oral alpha-blockers as tamsulosin 0.4mg, alfuzosin XL 10mg, doxazosin XL 8mg, terazosin 5mg or silodosin 8mg. The exclusion criteria were applied and all patients were divided into 2 groups. Group 1 (n=94) consisted of MtS patients and Group 2 (n=103) was consisted of patients without MtS. Demographic data including age, previous operations, comorbidities were recorded. The international prostate symptom score (IPSS), uroflowmetric parameters, post voiding residual urine volume (PVR), PSA, DRE, prostate volume and quality of life index (QoL) were noted. Anthropometric measurements including NC, WC, height, weight, body mass index (BMI) and blood pressure (BP) were recorded. Supine WC was measured at the level of the umbilicus with the patient breathing silently, and NC was measured with head erect and eyes facing forward, horizontally at the upper line of the laryngeal bulge as in the World Healh Organization guidelines ( 9 ). BP including systolic and diastolic was measured twice, with the second measurement taken 10 min. after the first. The means of BP measurements were recorded.

Blood analysis included FBG (mg/dL), TG (mg/dL), low-density lipid (LDL) (mg/dL), and HDL (mg/dL) and total cholesterol (mg/dL). All blood samples were obtained from the largest antecubital vein, after at least 12h of fasting in the morning. According to NCEP criteria the diagnosis of MtS included: BP ≥130/85mmHg, FBG ≥110mg/dL, WC ≥102cm, HDL-cholesterol <40mg/dL, TG ≥150mg/dL.

IPSS, PSA, maximum flow rate (Qmax) in uroflowmetry, and PVR were recorded before treatment as baseline. These parameters were compared in both groups at the 1 ^st^ month and 6 ^th^ month of medical treatment. Success rate was accepted when the IPSS decreased at least 4-6 points ( 12 ). The cut-off values were determined by using statistical analyses and the receiver-operating characteristic (ROC) curves were drawn. Besides, we evaluated response to alpha-blockers in terms of the cut-off values for NC and WC, in BPH patients with MtS.

### Statistical analyzes

The Statistical package for social sciences (SPSS) for Windows ver. 16.0 (SPSS Inc., Chicago, IL) was used for statistical analyzes and all graphs were provided by the same software program. The independent samples t-tests were employed to compare continuous data, and the One-way ANOVA analyzes of variance were also used for comparisons among groups. Statistical analyzes including ROC curves were performed; statistically significant p was accepted as p<0.05.

## RESULTS

Mean age was 60.5±8.1 years and mean BMI was 31.3±5.7kg/m ^2^ . The demographic data including IPSS, PVR, QoL index, PSA, prostate volume, and antropometric measurements were presented ( Table-1 ). In total, 197 patients (n=94 in Group 1 and n=103 in Group 2) were enrolled into the study. The baseline parameters areshown in Table-2 . Age, uroflowmerty parameters and QoL index were comparable between groups except BMI, NC, and WC were significant higher in Group 1 than Group 2 (p<0.001).

**Table 1:** Baseline demographic data in all patients (n=197).

Parameters	Mean±SD
Age (years)	60.5±8.1
Height (cm)	168.3±7.2
Weight (cm)	88.2±15
Neck circumference (cm)	39.4±4.4
BMI (kg/m ^2^ )	31.3±5.7
Waist circumference (cm)	104.4±14.9
Prostate volume (cc)	42.2±23.5
Triglyceride(mg/dL)	138.8±68.9
High Density Lipoprotein (mg/dL)	49.3±13.1
Systolic BP (mmHg)	116.2±18.7
Diastolic BP (mmHg)	74±13.2
Fasting blood glucose (mg/dL)	99.3±27.4
Qmax	15±4
Qavg	6.1±3
IPSS	23.4±4.1
PSA (ng/dL)	2.6±1.5
PVR (mL)	73.6±40.9
QoL score	3.5±1.6

Abbreviations: **BMI** = Body mass index; **BP** = Blood pressure; **IPSS** = International prostate symptom score; **Qavg** = Mean flow rate in uroflowmetry; **Qmax** = Maximum flow rate in uroflowmetry; **QoL** = Quality of life; **PSA** = Prostate specific antigen; **PVR** = Post voided urine volume

**Table 2:** Comparison of baseline parameters in groups.

Parameters	Group 1 (n=94)	Group 2 (n=103)	P Value
Age	59.6±8.3	61.3±7.8	0.14
BMI	35.2±4.4	27.7±4.3	<0.001*
Neck circumference	42.3±3.1	36.7±3.6	<0.001*
Waist circumference	114.2±11	95.4±12.3	<0.001*
Prostate Volume	42.9±22.5	41.5±24.5	0.67
Qmax	14.6±4.2	15.5±3.8	0.12
IPSS	23.4±3.8	23.5±4.8	0.84
PSA	2.7±1.6	2.6±1.5	0.67
PVR	77.3±39.1	70.2±40.3	0.22
QoL score	3.5±1.6	3.5±1.6	0.95

Abbreviations: **BMI** = Body mass index; **IPSS** = International prostate symptom score; **Qmax** = Maximum flow rate in uroflowmetry; **PSA** = Prostate specific antigen; **PVR** = Post voiding residual urine volume; **QoL** = Quality of life score

* Statistical significant P value

IPSS, maximum flow rate in uroflowmery (Qmax), PVR, and QoL were significant more developed in Group 2 than Group 1, at the 1 ^st^ month of oral alpha-blockers administration (respectively; p=0.03, p=0.03, p=0.04, p=0.005). These are presented in Table-3 . Similar significance was determined at 6 ^th^ month of medical treatment (respectively; p=0.02, p=0.03, p=0.04, p=0.001) ( Table-4 ).

**Table 3:** Comparison of uroflowmetry parameters and quality of life index between groups one month after treatment with alpha-blockers.

Parameters	Group 1 (n=94)	Group 2 (n=103)	P Value
**Qmax**	23.1±4	24.4±4.4	0.03*
**IPSS**	15.2±4.1	14±4.1	0.03*
**PVR**	66±32.8	56.1±37	0.04*
**QoL score**	5.1±1.5	5.7±1.5	0.005*

Abbreviations: **IPSS** = International prostate symptom score; **PVR** = Post voiding residual urine; **Qmax** = Maximum flow rate in uroflowmetry; **QoL** = Quality of life score.

*Statistical significant P value

**Table 4:** Comparison of uroflowmetry parameters and quality of life index between groups six months after treatment with alpha blockers.

Parameters	Group 1 (n=94)	Group 2 (n=103)	P Value
**Qmax**	23.1±4.3	24.6±4.9	0.02*
**IPSS**	15.2±4.1	13.9±4.2	0.03*
**PVR**	65.4±32.4	55.5±37	0.04*
**QoL score**	5.3±1.4	6.1±1.5	0.001*

Abbreviations: **IPSS** = International prostate symptom score; **PVR** = Post voiding residual urine; **Qmax** = Maximum flow rate in uroflowmetry; **QoL** = Quality of life score.

* Statistical significant P value.

Response to alpha-blockers (eloborated according to used drugs) were evaluated at baseline, 1 ^st^ and 6 ^th^ months of treatment by using Qmax, IPSS, PVR and QoL index ( Table-5 ). Silodosin was more effective than other drugs in all patients ( Table-5 ). In view of these, alpha-blockers were 80.9% successful in Group 1 (BPH patients with MtS) and 87.4% successful in Group 2 (BPH patients without MtS) (p<0.001). In total, success was 84.3% with alpha-blockers. Furthermore, according to statistical analyzes, silodosin was more succesful than other alpha-blockers in terms of developing Qmax, IPSS, PVR, and QoL parameters.

**Table 5:** Comparison of response to treatment according to used alpha-blockers.

Parameters	Silodosin 8mg (n=41)	Tamsulosin 0.4mg (n=39)	Alfuzosin XL 10mg (n=39)	Terazosin 5mg (n=38)	Doksazosin XL 8mg (n=40)	P value
Qmax mL/sec. at baseline	14.9±4.2	15±3.9	15±3.8	15.1±3.6	15.1±3.8	0.9
Qmax at 1 ^st^ month of treatment	25.9±4.8	24.23.8	23.4±3.5	22.7±4.6	22.5±3.7	0.002*
Qmax at 6 ^th^ month of treatment	26.4±5.1	24.3±4.5	23.3±3.9	22.6±4.6	22.5±4.2	<0.001*
IPSS at baseline	22.9±4.5	23.7±4.2	23.4±3.6	23.7±4.2	23.5±3.8	0.9
IPSS at 1 ^st^ month of treatment	11.7±3.8	14±3.4	14.8±3.5	16.1±4.3	16.5±4	<0.001*
IPSS at 6 ^th^ month of treatment	11.2±3.6	13.9±3.3	15.2±3.5	16±4.4	16.4±4.1	<0.001*
PVR at baseline	76.1±38.7	87.3±39	77.6±34.5	75.8±33.7	77.7±34.8	0.6
PVR at 1 ^st^ month of treatment	56.8±35.3	67.8±37.6	59.5±35	61.1±34.7	59.1±34.7	0.7
PVR at 6 ^th^ month of treatment	55.6±35.1	67.7±38.1	58.6±33.9	60.8±33.9	58.6±35.1	0.6
QoL at baseline	3.7±1.5	3.1±1.7	3.7±1.4	3.2±1.7	3.7±1.6	0.1
QoL at 1 ^st^ month of treatment	6.4±1.3	5.5±1.4	5.3±1.5	4.8±1.5	5±1.6	<0.001*
QoL at 6 ^th^ month of treatment	6.8±1.2	5.9±1.3	5.5±1.3	5.1±1.4	5.3±1.5	<0.001*

Abbreviations: **IPSS** = International prostate symptom score; **PVR** = Post voiding residual urine volume; **Qmax** = Maximum flow rate in uroflowmetry; **QoL** = Quality of life index.

* Statistical significant P value.

* One way Anova was used to compare values in groups.

**Figure 1:**
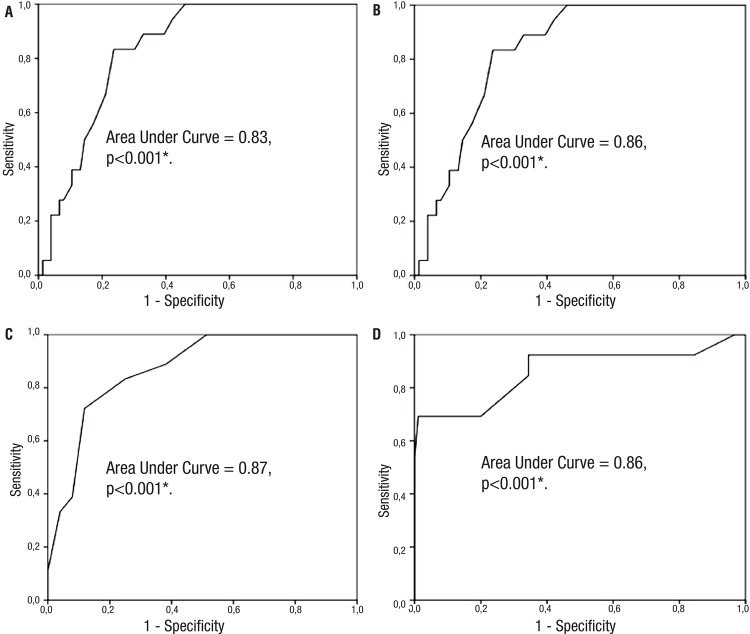
The cut-off values of neck circumference and waist circumference were drawn with Receiver-operating characteristic curves according to groups. A-The cut-off value for waist circumference in benign prostate hyperplasia patients with metabolic syndrome. B-The cut-off value for waist circumference in benign prostate hyperplasia patients without metabolic syndrome. C-The cut-off value for neck circumference in benign prostate hyperplasia patients with metabolic syndrome. D-The cut-off value for neck circumference in benign prostate hyperplasia patients without metabolic syndrome.

The ROC curves were obtained for determination of the cut-off values in terms of success of medical treatment with alpha-blockers. Mean WC was 113.5cm with 94.4% sensitivity and 42.1% specificity, in patients with metabolic syndrome. The area under the curve was “0.83”; p<0.001 ( Figure-1A ). Mean WC was 91.5cm with 92.3% sensitivity and 52.2% specificity, in Group 2. The area under the curve was “0.86”; p<0.001 ( Figure-1B ). On the other hand, the mean NC was 42.5cm with 88.9% sensitivity and 38.2% specificity, in Group 1 ( Figure-1C ). The area under the curve was “0.87”; p<0.001. Mean NC was 35.7cm with 92.3% sensitivity and 51.1% specificity, in Group 2. The area under the curve was “0.86”; p<0.001 ( Figure-1D ). In multivariate analyzes, MtS was considered the determinant factor for accurate response to alpha-blocker treatment. The cut-off value for NC was 42.5cm and for WC was 113.5cm for good response to alpha-blockers.

There was no remarkable side effect in both groups. Dizziness and asthenia were the most common side effects. On the other hand, 7 patients using silodosin and 2 patients using tamsulosin suffered unejaculation. However, all patients carried on medical treatments.

## DISCUSSION

MtS is accepted as one of the susceptibility factors for BPH ( 13 , 14 ). Besides, relationship between BPH and components of MtS including WC was earlier reported ( 15 ). The constituve parts of MtS were mentioned above and the WC is one of the essentials among them. WC also depicts visceral obesity. To measure WC sometimes can be very difficult, annoying, and time consuming, notably in the outpatient settings. Thus, NC can be used in these cases. Recently, NC has been reported as a useful diagnostic tool for determining visceral obesity ( 9 - 11 , 16 ). In the present study, we evaluated relationship between NC and BPH, in patients with MtS. Additionally, we showed low response to alpha-blockers in BPH patients with MtS. According to our best knowledge, this is the first study in the published literature on this issue including relationship between NC and BPH.

There were significant developments in IPSS, Qmax, QoL, and PVR with oral alpha-blocker treatments in both groups. However, the development was more significant in Group 2 than Group 1. In multivariate analyses, it was assumed MtS the determinant factor for dose response to alpha-blockers. In subgroup analyzes FDG, WC, and NC (with MtS) were more significant factors for dose response. In ROC curve analyzes, cut-off values were 113.5cm and 42.5cm for WC and NC, respectively, corresponding to good response to medical treatment in BPH patients with MtS. According to One-way-Anova analyzes, Silodosin was a promising molecule for improving lower urinary tract symptoms in MtS patients in terms of developed Qmax, IPSS, PVR and QoL. Roehrborn and Rosen reported increased QoL index with alfuzosin in BPH patients with MtS ( 17 ). Our findings were not parallel to their results. On the other hand, Kupelian et al. reported negative effect of MtS on dose response in BPH ( 18 ). Cyrus et al. concluded similar findings ( 5 ). Findings of the present study agreed with these studies. In our study, all alfa-blockers were effective but silodosin was more effective than other alpha-blockers. This fact may be associated with more selective effects of Silodosin. Nevertheless, oral alpha-blockers were more effective in Group 2 than Group 1. Additionally, NC was significantly shorter in Group 2 than Group 1. Low response to oral alpha-blockers may be caused by endothelial dysfunction, atherosclerosis-induced pelvic ischemia in MtS patients ( 19 ). Moreover, He et al. recently reported role of inflammation in MtS patients with BPH ( 20 ). Russo et al. pointed same issue that BPH and MtS were significant associated with high grade of inflammation scores including inversely related to intraprostatic heme oxygenase levels and increased metaflammation ( 21 ). However, we focused on clinical effects of alpha-blockers in BPH patients with MtS, more molecular based researches are needed for showing accurate pathway that may be subject of another future study in terms of determing more effective molecules in these patients settings.

DiBello et al. reported BPH and MtS association with elevated PSA levels and these could indirectly connect with decreased odds of having MtS and its components ( 22 ). On the other hand, Zoe et al. reported higher PSA levels in BPH patients with MtS ( 23 ). Our findings were not in the same line with DiBello et al. ( 22 ) but, were similar with results of Zou et al. ( 23 ). There was higher PSA levels in Group 1 than in Group 2 without statistical significance. Increased PSA may be caused by multifactorial reasons including inflammation in the first place ( 24 ). Demir et al. recently reported apoptosis index and inflammation during alpha-blocker usage ( 25 ). Besides, it is now well-known that both BPH and MtS includes inflammation ( 26 ). Inflammation associated with BPH and MtS can increase PSA levels. However, Alcaraz et al. reported that these associations may be related with prostate cancer formation ( 27 ). Nonetheless, there is need of much more well-designed studies for evidence based results on association between elevated PSA and prostate cancer in MtS patients ( 27 ).

There were also some side effects during alpha-blocker usage. Dizzeness was the most common one. However, there were no differences between groups for side effects. Additionally, none of the patients stopped the medical treatment. One of the annoying side effect was unejaculation which most occured with silodosin. This was not a reason to stop treatment.

There are some limitations in our study. Low number of patients in groups is one of them. Because of this, some statistical analyzes should be adequately interpreted: despite significant p values in Table-3 and 4 , the differences in all their parameters may really be not clinically significant. At this point, to define exact values of response to treatment may be difficult. Additionally, we did not research the molecular mechanism for relationship between MtS and BPH. The goals of the study were relationship betwen NC and BPH in patients with MtS. Also, the cut-off values for response to contemparary used alpha-blockers were showed, in ROC curves.

Finally, the association between BPH and MtS in terms of measuring WC and NC was presented. We could show that the presence of 43cm or higher NC could be associated with low response to alpha-blocker in BPH patients with MtS. Our results should be verified in future studies with a high number of patients. According to our best knowledge, this is the unique work in the published literature.

## CONCLUSIONS

MtS can be related to BPH and can negatively affect response to alphablocker treatment. NC can be used for predicting response to alpha-blocker treatment in MtS patients with BPH. NC of at least 43cm and/or above can be associated with low response to alpha-blocker treatment in patients with MtS. Thus, NC is a promising measurement that can show visceral obesity and response to medical treatment in BPH patients with MtS. More well-designed studies with high numbers of patients are needed for more accurate results on this issue.

## ARTICLE INFO

Int Braz J Urol. 2017; 43: 95-103
